# Inhibition of HIV-1 replication by P-TEFb inhibitors DRB, seliciclib and flavopiridol correlates with release of free P-TEFb from the large, inactive form of the complex

**DOI:** 10.1186/1742-4690-4-47

**Published:** 2007-07-11

**Authors:** Sebastian Biglione, Sarah A Byers, Jason P Price, Van Trung Nguyen, Olivier Bensaude, David H Price, Wendy Maury

**Affiliations:** 1 Interdisciplinary Molecular and Cellular Biology Program, University of Iowa, Iowa City, IA, USA; 2 Department of Microbiology, University of Iowa, Iowa City, IA, USA; 3 Department of Biochemistry, University of Iowa, Iowa City, IA, USA; 4 Laboratoire de Regulation de l'Expression Genetique, Ecole Normale Superieure, Paris, France; 5 CBR Institute for Biomedical Research, Harvard Medical School, Boston, MA, 02115, USA; 6 Oregon Health & Science University, Department of Molecular and Medical Genetics, Portland, OR 97239, USA

## Abstract

**Background:**

The positive transcription elongation factor, P-TEFb, comprised of cyclin dependent kinase 9 (Cdk9) and cyclin T1, T2 or K regulates the productive elongation phase of RNA polymerase II (Pol II) dependent transcription of cellular and integrated viral genes. P-TEFb containing cyclin T1 is recruited to the HIV long terminal repeat (LTR) by binding to HIV Tat which in turn binds to the nascent HIV transcript. Within the cell, P-TEFb exists as a kinase-active, free form and a larger, kinase-inactive form that is believed to serve as a reservoir for the smaller form.

**Results:**

We developed a method to rapidly quantitate the relative amounts of the two forms based on differential nuclear extraction. Using this technique, we found that titration of the P-TEFb inhibitors flavopiridol, DRB and seliciclib onto HeLa cells that support HIV replication led to a dose dependent loss of the large form of P-TEFb. Importantly, the reduction in the large form correlated with a reduction in HIV-1 replication such that when 50% of the large form was gone, HIV-1 replication was reduced by 50%. Some of the compounds were able to effectively block HIV replication without having a significant impact on cell viability. The most effective P-TEFb inhibitor flavopiridol was evaluated against HIV-1 in the physiologically relevant cell types, peripheral blood lymphocytes (PBLs) and monocyte derived macrophages (MDMs). Flavopiridol was found to have a smaller therapeutic index (LD_50_/IC_50_) in long term HIV-1 infectivity studies in primary cells due to greater cytotoxicity and reduced efficacy at blocking HIV-1 replication.

**Conclusion:**

Initial short term studies with P-TEFb inhibitors demonstrated a dose dependent loss of the large form of P-TEFb within the cell and a concomitant reduction in HIV-1 infectivity without significant cytotoxicity. These findings suggested that inhibitors of P-TEFb may serve as effective anti-HIV-1 therapies. However, longer term HIV-1 replication studies indicated that these inhibitors were more cytotoxic and less efficacious against HIV-1 in the primary cell cultures.

## Background

During HIV-1 replication, the host polymerase (Pol II) is recruited to the viral promoter within the long terminal repeat (LTR) and initiates transcription [1]. Pol II initiates transcription, but elongation of most of the transcripts is blocked by negative elongation factors [2,3]. The HIV-1 transcription transactivator Tat binds to the bulge of the HIV-1 RNA stem loop termed TAR that is found in all nascent HIV-1 messages and recruits positive transcription elongation factor b (P-TEFb) to the LTR [reviewed in [4,5]]. P-TEFb phosphorylates both the carboxyl-terminal domain (CTD) of Pol II [6] and the negative elongation factors [2,7] allowing Pol II to transition from abortive to productive elongation [8].

P-TEFb is found within a cell in two forms referred to as large and free forms [9,10]. The kinase active, free form contains Cdk9 and one of several cyclin regulatory subunits, cyclin T1, cyclin T2a, cyclin T2b or cyclin K, with cyclin T1 being the predominantly associated cyclin in many cell types [11,12]. The kinase inactive, large form of P-TEFb additionally contains 7SK RNA [9,10] and hexamethylene bisacetamide-induced protein 1 (HEXIM1) [13,14] or HEXIM2 [15]. In HeLa cells, between 50% and 90% of P-TEFb is present in the large form of the complex while the remainder of P-TEFb is in the kinase active, free form [9,10,14,15]. It is hypothesized that the large form of P-TEFb serves a reservoir for the free form.

All currently approved anti-HIV therapies target viral proteins that have been shown to rapidly evolve under the selective pressure of highly active anti-retroviral therapy (HAART) [16-18]. Mutations in the viral genome that decrease the effectiveness of HAART arise as a result of the selection of random mutations generated by the lack of proofreading activity in HIV reverse transcriptase [17,19] and by G to A hypermutation that is believed to result from APOBEC3G restriction [20]. Thus, identification and characterization of additional anti-virals is a necessity. Anti-virals against cellular targets that are required for virus replication may prove to be highly effective. Furthermore, evolution of HIV resistance to this group of compounds might be less likely. Consistent with this possibility, an extensive 6 month study aimed at generating a HIV-1 strain resistant to the cyclin-dependent kinase inhibitor, roscovitine, proved unsuccessful [21].

Targeting P-TEFb kinase activity as an anti-HIV therapy is potentially attractive, but has not been extensively evaluated. The P-TEFb inhibitors DRB and flavopiridol have been demonstrated to effectively inhibit HIV Tat-dependent transcription in cell lines [22,23]. Limited studies of the effect of these inhibitors on HIV replication demonstrate a significant reduction of replication at concentrations with limited cytotoxicity [22,23]. The anti-retroviral activity of roscovitine or the R-enantiomer of roscovitine (seliciclib or Cyc202) has also been explored. This inhibitor has a spectrum of inhibitory activities against a number of cyclin dependent kinases including Cdk 1, 2, 7 and 9 [24]. A previous examination of the effect of seliciclib on HIV replication had focused on its inhibition of Cdk2 activity [25].

The use of P-TEFb inhibitors as chemotherapeutic agents against cancers has also been proposed [26]. Flavopiridol and seliciclib showed modest cytotoxicity when tested in clinical trials against different kinds of cancers [reviewed on [27]]. In phase II cancer clinical trials, fatigue, venous thromboses and diarrhea were the primarily side effects of flavopiridol infusions that achieved plasma flavopiridol levels of approximately 400 nM during a 72 hour treatment period [28-31]. Phase II monotherapy trials with flavopiridol have proved disappointing [30] and newer studies have combined flavopiridol with other chemotherapeutic agents [32,33]. Seliciclib has recently been tested as a chemotherapeutic agent in Phase I trials and was shown to cause fatigue and elevated creatinine at the highest tested doses that achieved maximal plasma levels of 2 to 4 μg/ml [24,34].

In this study, we sought to characterize the anti-HIV activity of the cyclin-dependent kinase inhibitors DRB, flavopiridol and seliciclib. In HeLa cells, we found that the anti-HIV activity of these compounds correlated with concentrations that released free P-TEFb from the large form of the complex. These concentrations were not cytotoxic to cells despite the known requirement of P-TEFb activity for Pol II-dependent transcript elongation. However, the concentration of these compounds that was needed to inhibit HIV replication in PBLs and MDMs was higher. Compound cytotoxicity was also greater in these primary cells decreasing the likely utility of these compounds in controlling HIV replication in infected individuals.

## Results

### Inhibition of Cdk9 kinase activity by P-TEFb inhibitors

**Figure 1:**
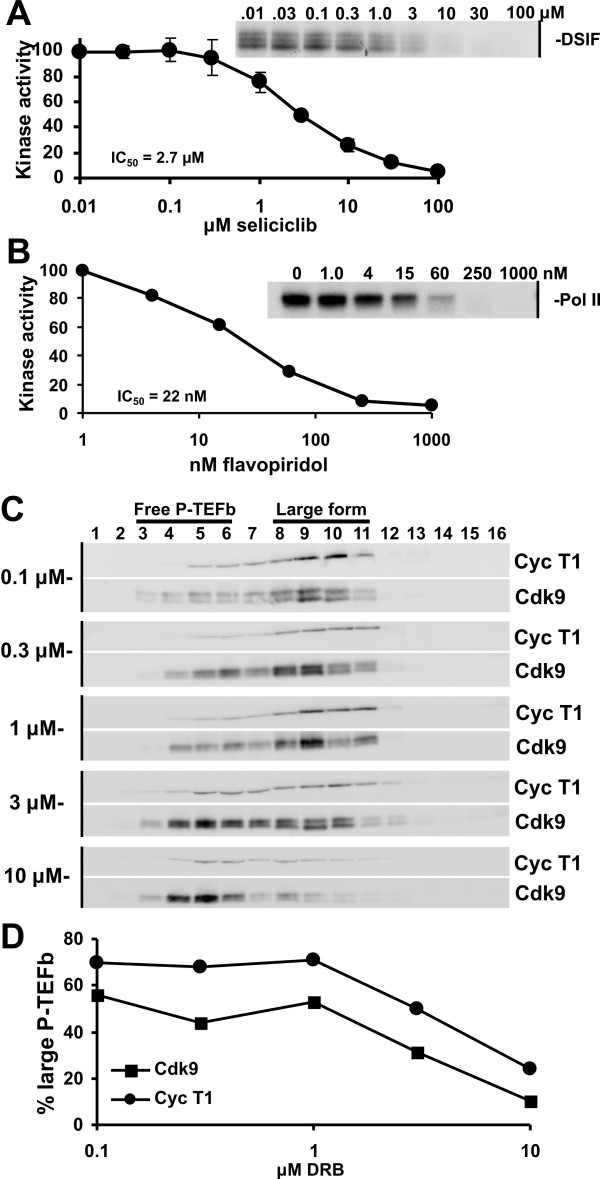
Effects of P-TEFb inhibitors on the kinase activity of P-TEFb i*n vitro *and on the large form of P-TEFb in cells. *In vitro *P-TEFb kinase assays were performed using recombinant P-TEFb, Pol II CTD or DSIF in the presence of increasing concentrations of seliciclib (A) or flavopiridol (B). The kinase reactions were resolved by SDS-PAGE and the amount of incorporated γ-^32^P-ATP was quantitated with a Packard InstantImager. (C and D) Glycerol gradient analysis of HeLa37 cells treated with DRB. (C) HeLa37 cells were treated with increasing amounts of DRB for 1 hour and lysed to extract both forms of P-TEFb from the nucleus. The lysates were subjected to glycerol gradient sedimentation and the fractions were examined by immunoblotting for Cdk9 and cyclin T1. (D) The Cdk9 and cyclin T1 signals in the free (fractions 3–6) and large (fractions 8–11) forms of P-TEFb were calculated and plotted as a function of the concentration of DRB used in the cell treatment.

To determine the effectiveness of preparations of the cyclin dependent kinase inhibitors, flavopiridol and seliciclib, we performed *in vitro *kinase assays with recombinant P-TEFb. As expected, increasing concentrations of the P-TEFb inhibitors also decreased phosphorylation of the protein substrate. Phosphorylation of the largest subunit of DSIF by P-TEFb was inhibited by concentrations of seliciclib of 1 μM or higher, and an IC_50 _of 2.7 +/- 0.4 μM was determined (Fig. 1A). Phosphorylation of the CTD of the largest subunit of Pol II was inhibited by low concentrations of flavopiridol and for this drug under the conditions used an IC_50 _of 22 nM was calculated (Fig. 1B). The preparation of DRB was tested earlier and an IC_50 _of 0.9 μM was found [11]. These results indicate that the three compounds perform in a manner consistent with other published studies. The absolute IC_50_'s determined i*n vitro *using kinase assays should not be compared to IC_50_'s for the effects of the compounds *in vivo*. This is because, except for flavopiridol, all P-TEFb inhibitors are competitive with ATP and therefore the absolute IC_50_'s are dependent on the ATP concentration [22]. Because flavopiridol binds one to one with P-TEFb even at sub-nanomolar levels, the IC_50 _for inhibition of P-TEFb by flavopiridol is dependent on the concentration of P-TEFb [22].

### Treatment of cells with DRB leads to release of P-TEFb from the large form

To examine the effect of DRB treatment of cells, we treated HeLa cells with increasing concentrations of DRB and analyzed the quantity of large and free forms of P-TEFb within the cell 1 hour later. Glycerol gradient fractionation of lysates followed by immunoblotting of the fractions has been shown to reproducibly separate the forms of P-TEFb with the larger molecular weight form sedimenting with higher concentrations of glycerol than the free form [10,15,35]. Quantitative analysis of the immunoblots provides an accurate representation of the ratio of large to free form of P-TEFb in cells. Increasing concentrations of DRB resulted in a shift in the ratio of P-TEFb forms (Fig. 1C). In the absence of DRB, approximately 60% of Cdk9 and 70% of cyclin T1 were located in the denser fractions (fractions 8–11) containing the large form of P-TEFb. In the presence of the highest concentration of DRB tested, 10 μM, only about 20% of P-TEFb subunits were left in the large form of the complex. By plotting the quantity of Cdk9 and cyclin T1 present in the large form of P-TEFb in the presence of DRB, it was observed that approximately 3 μM DRB caused a 50% reduction in large form within the cell (Fig. 1D). This gradual release of P-TEFb from the large form as DRB was increased suggests that the cells are trying to compensate for the loss of P-TEFb activity by releasing more active P-TEFb from the large form.

### The free and large forms of P-TEFb are extracted from cell nuclei at different ionic strengths

We were interested in determining if a similar correlation between kinase inhibition and loss of large P-TEFb was found in cells treated with other P-TEFb inhibitors. However, glycerol gradient sedimentation studies require large numbers of cells and are reagent and time intensive. The development of an efficient and rapid method to examine the ratio of large to free form of P-TEFb would allow for the examination of small populations of cells or the simultaneous characterization of many treatments.

**Figure 2:**
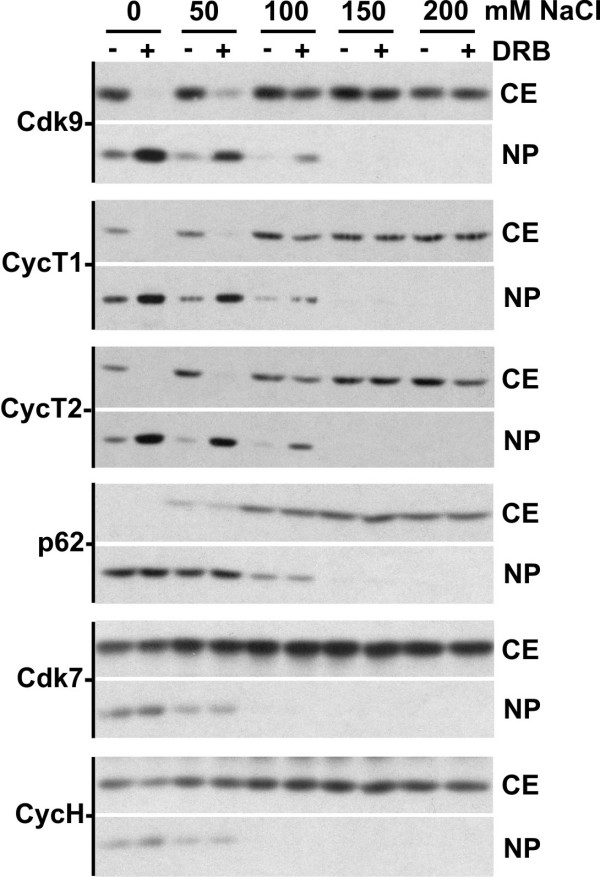
Characterization of P-TEFb retention by HeLa cell nuclei using differential salt extraction. Untreated HeLa cells and HeLa cells treated for 1 hour with 100 μM DRB were lysed with a buffer containing the indicated amounts of NaCl to generate cytosolic extracts (CE). The CE and the nuclear pellet (NP) were examined by immunoblotting with the indicated antibodies for the presence of P-TEFb or the TFIIH components p62, Cdk7 and cyclin H.

To determine if the two forms of P-TEFb could be separated easily we examined the extractability of P-TEFb from nuclei of detergent treated cells. The retention of P-TEFb in the nuclear pellet (NP) was examined in untreated and 100 μM DRB-treated cells lysed with buffers containing increasing concentrations of NaCl (Fig. 2). Approximately 50% of Cdk9, cyclin T1 and cyclin T2 were present in cytosolic extracts (CE) prepared from untreated cells that were lysed under low salt conditions. Identical conditions have been demonstrated by glycerol gradient sedimentation analysis to extract only the large form of P-TEFb [36]. P-TEFb subunits were not detected in cytosolic extracts of DRB-treated cells prepared with the same low salt lysis buffer. As the salt in the lysis buffer was increased, the amount of P-TEFb present in the cytosolic extract was increased in both the untreated and the DRB-treated cells. One hundred and fifty millimolar NaCl extraction conditions have been demonstrated to yield both form of P-TEFb as detected by glycerol gradient sedimentation analysis [15]. As controls, the differential salt extractability of the TFIIH subunits p62, Cdk7 and cyclin H were also examined. The salt extractability of Cdk7, cyclin H and p62 was unaffected by the addition of DRB and, consistent with their association with chromatin, increasing concentrations of these proteins were found in the cytosol fraction with increasing concentrations of salt. Taken together, these data indicated that the free and large forms of P-TEFb have differential salt extractability from nuclei, with the large form present in the cytosolic fraction under low salt conditions and the free form requiring more than 100 mM NaCl to be completely extracted. Additionally, the loss of large form within the cell in the presence of high concentrations of P-TEFb inhibitors was demonstrated by the persistence of more of the P-TEFb remaining in the nuclear pellet when lysis buffers containing 100 mM NaCl or less were used for extraction. We tentatively conclude that differential salt extraction separates the free and large forms of P-TEFb. Retention in the nucleus of P-TEFb that is not bound to HEXIM1 and 7SK under very low salt conditions is presumably due to its salt-sensitive interaction with chromatin associated proteins, such as Brd4 [37,38], and other DNA bound transcription factors [8].

### P-TEFb inhibitors shift the ratio of free to large P-TEFb forms in cells

**Figure 3:**
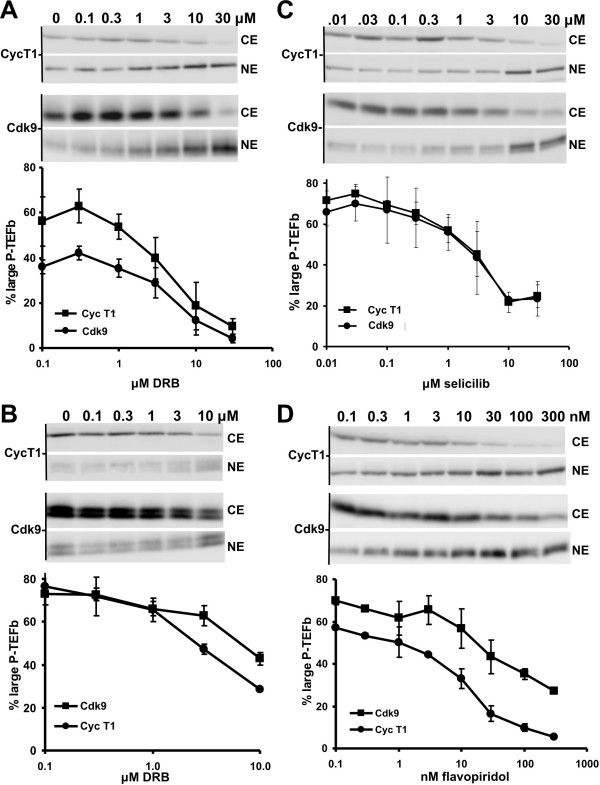
The P-TEFb inhibitors DRB, seliciclib and flavopiridol release P-TEFb from the large form. Low-salt cytosolic extract (CE) containing the large form of P-TEFb and high-salt nuclear extracts (NE) containing the free form of P-TEFb were generated from (A) DRB-treated HeLa cells, (B) DRB treated Jurkat cells, (C) seliciclib-treated HeLa37 cells or (D) flavopiridol-treated Jurkat cells. Quantitative western blotting was performed on low salt cytosolic extracts (CE) and high-salt nuclear extracts (NE) to detect the percentage of Cdk9 and cyclin T1 present in the free and large form of the P-TEFb complex. The percent of P-TEFb in the large form of the complex (low-salt or CE) was calculated as a fraction of the total amount of P-TEFb (low-salt + high-salt P-TEFb) and plotted as a function of the concentration of P-TEFb inhibitor.

If the amount of large and free forms of P-TEFb were accurately reflected by our novel salt extraction assay, we would anticipate that using this assay with increasing concentrations of P-TEFb inhibitors would give similar dose response curves to those obtained in our glycerol gradient studies. HeLa37 cells were treated with the indicated amounts of DRB for 1 hour and lysed with the low salt buffer to generate cytosolic extracts containing the large form of P-TEFb and a nuclear pellet containing the free form of P-TEFb. Free P-TEFb was eluted from the nuclear pellet by extraction with a buffer containing 450 mM NaCl. The cytosolic extract (CE) and the nuclear extract (NE) were analyzed by western blotting for the presence of Cdk9 and cyclin T1 (Fig. 3A). In untreated HeLa37 cells, approximately half of the P-TEFb was present in the cytosolic extract. As the concentration of DRB was increased, the fraction of P-TEFb in the cytosolic extract decreased while the fraction of P-TEFb in the nuclear extract increased. The IC_50 _for the release of free form of P-TEFb from the large form of the complex by DRB was about 4.5 μM (Fig. 3A), a concentration of DRB similar to that found in our glycerol gradient studies to release 50% of large P-TEFb. We conclude that the differential salt extraction assay can be used to determine the relative abundances of the two forms of P-TEFb. A similar study was carried out using DRB-treated Jurkat cells. Although the starting level of large form was higher (75 to 80%), a gradual reduction of the large form was seen at similar concentrations of DRB (Fig. 3B).

Using the newly developed assays, we next determined if seliciclib and flavopiridol also caused a release of P-TEFb from the large form of the complex. Treatment of HeLa37 cells with seliciclib (Fig. 3C) or Jurkat cells  with flavopiridol (Fig. 3D) led to a gradual reduction in the amount of the large form of P-TEFb. The IC_50_'s calculated for the transitions are summarized in Table 1. The concentrations needed to elicit release of half of the large form correlated with the strength of the P-TEFb inhibitor with flavopiridol being the most potent and DRB and selecilib being similar to each other.

**Table 1:** Summary of results

**Assay**	**DRB (μM)**	**Seliciclib (μM)**	**Flavopiridol (nM)**
IC_50 _of kinase inhibition:	0.9*	2.7	22
IC_50 _of loss of large form from HeLa cells:			
Cyclin T1	5 +/- 3	2.7 +/- 1	12 +/- 4
Cdk9	4.2 +/- 1.6	3.2 +/- 1.4	26 +/- 6
IC_50 _of inhibition of HIV in HeLa 37 cells:	2.6	3	9.5
Hela37 cell cytotoxicity (LD_50_):	20	12.5	225
Hela37 therapeutic index:	7.7	4.2	23.7
PBL therapeutic index:	N/D	N/D	2.7
MDM therapeutic index:	N/D	N/D	1.7

* as described in Peng et al (1998) Genes and Dev. 12: 755–762.

N/D = not determined

### Inhibition of HIV-1 infection by non-cytotoxic concentrations of P-TEFb inhibitors

**Figure 4:**
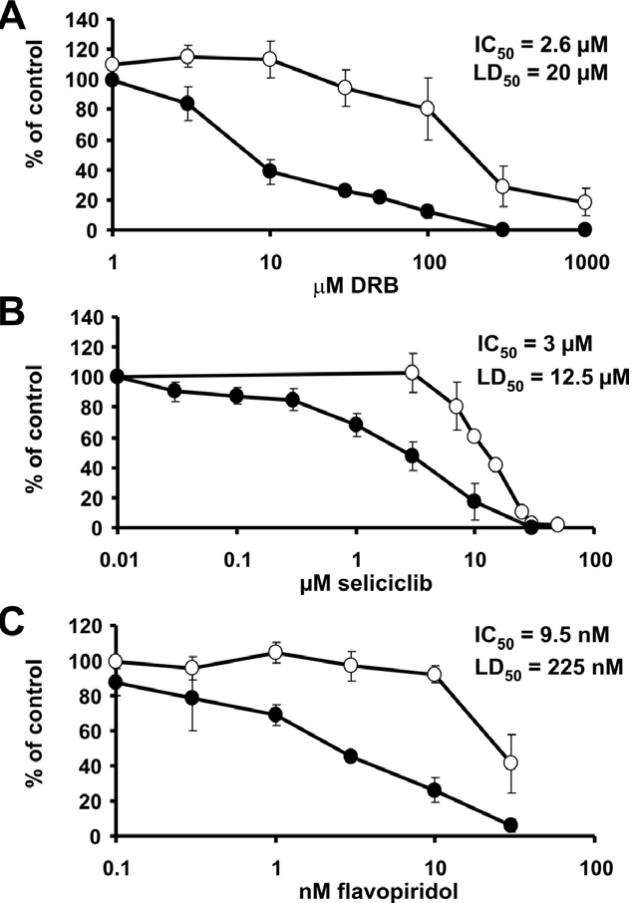
Inhibition of HIV-1 infectivity by non-cytotoxic concentrations of the P-TEFb inhibitors DRB, seliciclib and flavopiridol. HeLa37 cells were infected with HIV-1_p256 _and treated with the indicated amounts of (A) DRB, (B) seliciclib and (C) flavopiridol. After 40 hours the cells were fixed, immunostained for HIV antigens and the number of HIV positive cells counted. The number of infected cells (solid circles) was normalized to the control infection and plotted. Cell viability studies were performed in parallel. Values form cytotoxicity studies (open circles) were normalized to the mock treated cells and plotted.

To determine the impact of the P-TEFb inhibitors DRB, flavopiridol and seliciclib on HIV infectivity, single-round HIV-1 infectivity assays in HeLa37 cells were performed in the presence of increasing concentrations of inhibitors. HeLa37 cells that express CD4 and CCR5 as well as endogenous CXCR4 were infected with HIV in the presence of the P-TEFb inhibitors. Cells were fixed at 40 hours following initiation of the experiment and immunostained for expression of HIV antigens. The number of HIV-1 infected cells in each well was enumerated and dose response curves for the P-TEFb inhibitors were determined (Fig. 4). Studies measuring cytotoxicity of the inhibitors were performed in parallel. From the dose response curves, concentrations of inhibitors that decreased virus infection by 50% (IC_50_) as well as the concentration that resulted in a 50% decrease in cell viability (LD_50_) were determined. The IC_50 _for inhibition of viral infection in HeLa37 by DRB was 2.6 μM whereas the LD_50 _of DRB was 20 μM, yielding a therapeutic index (T.I. = LD_50_/IC_50_) of 7.7 (Fig. 4A and Table 1). Seliciclib exhibited an IC_50 _of 3 μM and an LD_50 _of 12.5 μM (Fig. 4B) generating the smallest therapeutic index of the three P-TEFb inhibitors tested at 4.2. The T.I. of flavopiridol was 23.7 as its IC_50 _was 9.5 nM and its LD_50 _was determined to be 225 nM (Fig. 4C). Concentrations of each of the P-TEFb inhibitors that inhibited HIV-1 replication correlated well with concentrations that caused a release of P-TEFb from the large complex. The concentrations of P-TEFb inhibitor that were cytotoxic to HeLa cells were 4 to 24 fold higher. These findings were indicative of the sensitivity of HIV transcription to loss of cellular P-TEFb activity and are consistent with previous observations [22,23]. The close correlation between the loss of the large form of P-TEFb in the cell and the reduction of HIV infectivity demonstrates the tight regulation of the kinase activity in cells and the absolute requirement of that activity for HIV replication. Hence, our findings suggested that the P-TEFb inhibitor flavopiridol that gave the largest therapeutic index value might serve as a promising anti-viral against HIV-1.

### Flavopiridol inhibits long-term HIV-1 replication in PBLs and MDMs

**Figure 5:**
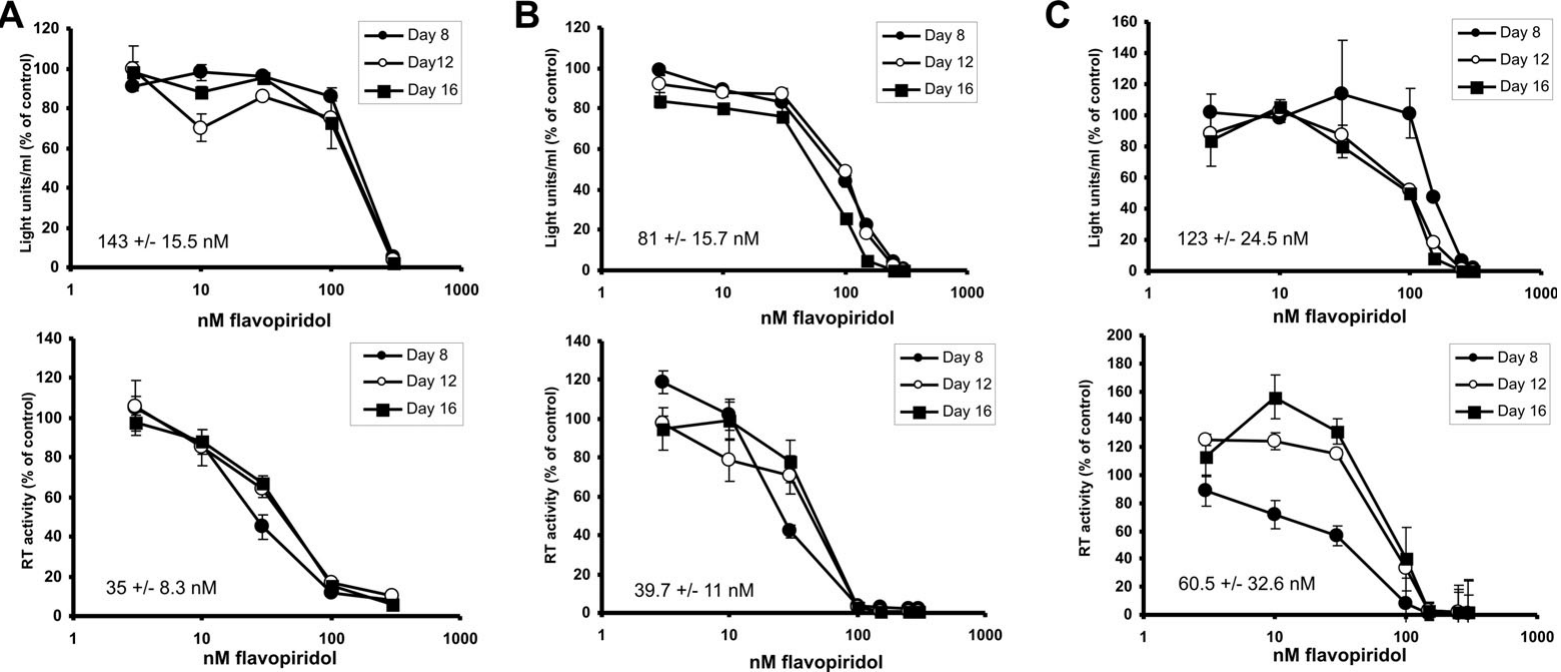
Inhibition of HIV replication in PBLs by flavopiridol. Isolated PBLs from three independent donors (A, B and C) were infected with HIV-1_p256 _and treated with increasing concentrations of flavopiridol. Supernatants were collected at 4, 8, 12 and 16 days post-infection. The amount of HIV-1 infection was measured by quantifying the amount of HIV-1 reverse transcriptase enzyme (RT) in the supernatants on the indicated days (BOTTOM graph for of each panel). Cytotoxicity studies were performed on uninfected PBLs by treating cells with increasing concentrations of flavopiridol for 4, 8, 12 and 16 days. Cell viability was estimated by performing ATPLite assay. The light readings were normalized to the mock treated cells and plotted (TOP graph for each panel).

To determine if HIV replication was blocked by P-TEFb inhibitors in clinically relevant cells, HIV-1 infectivity studies were performed in peripheral blood lymphocytes (PBLs) and monocyte-derived macrophages (MDMs) in the presence of increasing concentrations of flavopiridol. Flavopiridol was selected for this study not only because it demonstrated the best therapeutic index, but also because it had previously been shown to cause little to no inhibition of cellular transcription at low nanomolar concentrations [39]. PBLs were isolated from three different, healthy, HIV negative donors and activated with PHA and IL-2 prior to infection with 10,000 RT units of the dual-tropic strain of HIV-1_p256_. HIV-1 infected PBLs were treated with different concentrations of flavopiridol (0.1 to 1000 nM) for a period of 16 days with refreshed media and flavopiridol every 4 days. Cell viability studies were performed to determine the cytotoxic effects of flavopiridol in PBLs and supernatants were collected on days 4, 8, 12 and 16 and analyzed for the presence of the HIV RT enzyme (Fig. 5). The amount of RT activity or cytotoxicity in PBL cultures in the presence of flavopiridol was normalized to the untreated control values for all time points to allow comparisons between the different time points and inhibitor concentrations. Dose response curves were generated and IC_50 _and LD_50 _values were calculated for each time point and averaged together at the end of the experiment. The average IC_50 _for inhibition of viral replication in the presence of flavopiridol for donor #1 was 35 nM and the LD_50 _was 143 nM, yielding a T.I. of 4.1 (Fig. 5A and Table 1). Donor #2 (Fig. 5B) and donor #3 (Fig. 5C) exhibited IC_50 _values of 40 nM and 61 nM respectively while their LD_50 _values were 81 nM for donor #2 and 123 nM for donor #3. The T.I. for donors #2 and #3 were both approximately 2. These data showed that flavopiridol was able to inhibit HIV replication in PBLs but with reduced efficacy. Furthermore, long term culture of the primary cells in the P-TEFb inhibitor resulted in increased cytotoxicity (Table 1).

**Figure 6:**
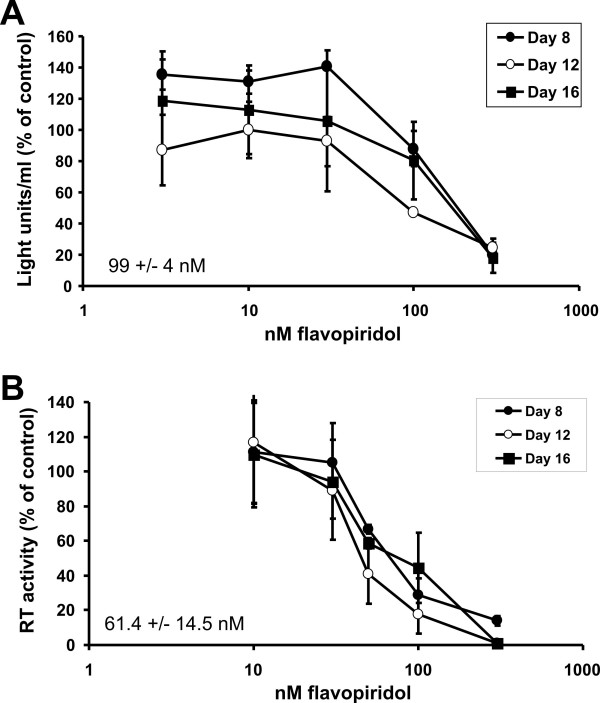
Inhibition of HIV-1 replication in MDMs by flavopiridol. MDMs were isolated from healthy donors and infected with HIV-1_p256 _along with increasing concentrations of flavopiridol. Supernatants were collected at 4, 8, 12 and 16 days post infection. The amount of HIV-1 infection was measured by quantifying the amount of HIV reverse transcriptase enzyme (RT) in the supernatants on the indicated days (BOTTOM graph). Cytotoxicity studies were performed on uninfected MDMs by treating cells with different concentrations of flavopiridol for 4, 8, 12 and 16 days and measuring cell viability by the ATPLite assay. The light readings were normalized to the mock treated cells and plotted (TOP graph). The experiment was performed in MDMs twice and the data from both experiments was pooled, averaged and graphed.

Similar HIV inhibition studies were performed with flavopiridol in monocyte derived macrophages (MDMs). The yield of MDMs from a single blood donor was about 10 fold lower than that of PBLs, limiting the number of independent replicas per experiment that could be performed. To obtain an accurate IC_50 _for inhibition of HIV replication by flavopiridol in MDMs, data from independent donors were pooled and analyzed. The average IC_50 _value for inhibition of HIV-1 replication by flavopiridol was 61 nM and the LD_50 _value was determined to be 99 nM, yielding a T.I. of 1.7 (Fig. 6). Thus, while flavopiridol did inhibit HIV replication in primary cells, flavopiridol inhibition was not as effective in these longer term assays as in the short term, single hit infectivity assays. In addition, these clinically relevant cells appeared to be more sensitive to the cytotoxic effects of flavopiridol reducing the therapeutic window of this compound.

## Discussion

Here, we developed a new approach for determining the ratio of large to free form of P-TEFb based on the differential salt extractability of the two forms of the complex. The differential salt extractability of the free and large P-TEFb forms provides a simple and rapid method for the separation of the two forms. We used differential salt extraction to demonstrate that P-TEFb inhibitors caused a dose dependent release of P-TEFb from its inactive, large form. Importantly, ability of HIV-1 to replicate in short term assays correlated with the amount of the large form of P-TEFb remaining. Findings from short term infectivity studies suggested that these P-TEFb inhibitors might be effective anti-HIV therapies. In these assays, flavopiridol had the most promising therapeutic index against HIV-1. However, in longer term HIV-1 replication assays in primary cells the IC_50 _values for flavopiridol inhibition were higher than those found in the short term assays and an increase in cytotoxicity reduced the T.I. in MDMs to less than two.

HAART regimens currently target viral proteins including the HIV-1 protease and reverse transcriptase enzymes [40]. One of the problems that HAART therapy faces is the development of resistant strains of HIV-1 that arise due to a high rate of viral mutation. A possible advantage of targeting a cellular protein such as P-TEFb is to avoid the generation of drug-resistant strains of virus. The limited studies performed to date with roscovitine suggest that resistant viruses against this kinase inhibitor may arise slowly if at all in tissue culture [21]. Including a P-TEFb inhibitor in HAART would decrease Tat-dependent transcription while potentially leading to a lower incidence of drug-resistant strains of HIV due to the stringent requirement of cellular P-TEFb for productive HIV-1 transcription [41-43]. Thus, we investigated the efficacy of three P-TEFb inhibitors against HIV-1.

Our studies demonstrate that the P-TEFb inhibitors DRB, flavopiridol and seliciclib inhibit HIV-1 infectivity in HeLa37 cells and to a lesser extent in longer replication studies performed in PBLs and MDMs. The IC_50 _of 9.5 nM for flavopiridol inhibition obtained during our single-round infectivity studies was consistent with the previously reported inhibition of HIV-1_HXB2 _infection in Sx22-1 indicator cells [22]. Likewise, the IC_50 _and LD_50 _values we obtained for DRB were similar to previously reported values on inhibition of Tat-dependent transcription [23] and for the inhibition of virus replication by seliciclib [23,25].

The P-TEFb inhibitor flavopiridol blocked HIV replication in MDMs and PBLs with a lower therapeutic index than that found in HeLa37 cell studies due to both a higher IC_50 _and lower LD_50 _values. Flavopiridol is the most effective and specific P-TEFb inhibitor currently identified [22,39,44] and yet efficacy of flavopiridol against HIV at non-cytotoxic concentrations is not promising. While clinical chemotherapeutic trials achieved transient plasma concentrations of flavopiridol 8 to 10 fold higher than the anti-viral IC_50 _values we obtained in primary cells, our findings suggest that consistent plasma levels of flavopiridol of 100 nM or higher would be needed to effectively impact HIV-1 replication and maintaining such levels would be associated with unacceptable levels of toxicity.

Treatment of cells with P-TEFb inhibitors shifted the ratio of free to large form of P-TEFb within cells as the inhibitors blocked kinase activity. P-TEFb may be released from the large form of the complex to compensate for the loss of P-TEFb activity. The P-TEFb inhibitor-induced reduction in the amount of large P-TEFb correlated with inhibition of viral replication suggesting the possibility that large P-TEFb is necessary for HIV-1 replication. Consistent with this possibility, a recent study indicates that HIV-1 Tat is able to recruit P-TEFb out of the large form thereby reducing the quantity of large form within HIV-1 infected cells [45]. Alternatively, the large form of P-TEFb may not be required for viral replication. Instead, specific levels of P-TEFb activity within the cell maybe critical for HIV transcription. Thus, eliminating the kinase activity reduces HIV-1 transcription in parallel. In this model, the total amount of P-TEFb kinase activity required for HIV-1 replication is greater than that needed for cellular transcription and release of P-TEFb from the large form is not sufficient to compensate for the inhibitor-induced loss in Cdk9 activity. To address the role of the large form of P-TEFb in HIV replication, a previous study reduced 7SK levels within the cell by siRNA causing a reduction in large P-TEFb [46]. The reduction in the quantity of large form of P-TEFb did not affect HIV-1 transcription and replication [46]. This finding suggests that the large form of P-TEFb does not play a critical role in HIV-1 transcription and that alterations in the fine balance of kinase active P-TEFb within the cell is responsible for the loss of HIV replication.

Finally, the development of a new salt extraction method that allows separation of large and free P-TEFb may prove useful for future studies. This assay is both less laborious than glycerol gradient fractionation and requires fewer cells. The differential salt extractability of P-TEFb is presumably based on the tight association that free P-TEFb has with chromatin [47-51]. The large form of P-TEFb may be untethered and free to move about the nucleus, perhaps to deliver P-TEFb to where it is needed while maintaining Cdk9 in its inactive state would minimize off target phosphorylations. Alternatively, since P-TEFb can localize to nuclear speckles [52], differential salt extractability might be due to differential localization of the large and free forms of the complex. This latter alternative is unlikely since P-TEFb localization is not altered by DRB treatments that completely inhibit transcription and disrupt large form of P-TEFb [52]. Therefore, we propose that the free form of P-TEFb is retained in the nucleus under low salt conditions due to its involvement in transcription and association with chromatin through numerous interactions with transcription factors [47-51]. All inhibitors of Pol II elongation that have been tested (flavopiridol, DRB, actinomycin D, ultraviolet irradiation) cause the release of P-TEFb from the large form, but the mechanism of release of is not currently understood [8]. Future studies aimed at uncovering mechanistic details of this process would be facilitated by the new method described here.

## Conclusion

Here, we developed a rapid assay that allowed us to quantitatively determine the amount of large and free forms of P-TEFb present in cells. Using this assay, we found that three P-TEFb inhibitors reduced the amount of the large form of P-TEFb in a dose dependent manner. Furthermore, initial short term studies with P-TEFb inhibitors demonstrated that loss of the large form of P-TEFb correlated with a reduction in HIV-1 infectivity without significant cytotoxicity. HIV-1 replication studies in primary cell cultures indicated that these inhibitors were more cytotoxic and less efficacious against HIV-1. How effective P-TEFb inhibitors would be at blocking HIV-1 replication *in vivo *is not clear.

## Methods

### Cell lines

HeLa S3 and HeLa37 cells (which exogenously express CD4 and CCR5) [53] were grown in DMEM with 10% fetal calf serum (FCS) and 1% penicillin/streptomycin. HeLa37 cells were a gift from Dr. David Kabat (Oregon Health & Science University, Portland, OR). Jurkat cells (ATCC #TIB 152) were grown in RPMI with 10% fetal calf serum and 1% penicillin/streptomycin. All cells were grown at 37°C and 5% CO_2_.

### Compounds and antibodies

DRB was obtained from Sigma and resuspended in ethanol to generate a 10 mM stock solution. Seliciclib (R-roscovitine) was obtained from Cyclacel (Dundee, Scotland, UK) and resuspended in DMSO to generate 10 mM stock solutions. Flavopiridol was obtained from NIH AIDS Research and Reference Reagent Program (Cat. #9925) and diluted in DMSO to generate a 10 mM stock solution. All compounds were aliquoted and stored at -80°C. Anti-Cdk9 (T-20), anti-cyclin T1 (T18) and anti-cyclin T2 rabbit polyclonal antibodies were obtained from Santa Cruz. Anti-Cdk7, anti-cyclin H and anti-p62 mouse monoclonal antibodies were a kind gift from J.M. Egly (Strasbourg, France).

### Generation of HIV

Virus generated from the dual-tropic molecular clone of HIV-1_p256 _[54] was used through out this study. p256 contains the V3 region from a patient isolate inserted into HIV-1_pNL4-3 _backbone [54]. 293T cells were seeded at 5 × 10^5 ^cells per well in a six-well tray a day before transfection. Cells were transfected with 7 μg of p256 proviral DNA expressing plasmid using the calcium phosphate procedure to generate HIV-1_p256 _viral stocks [53]. Virus-containing supernatants were collected at 24, 48, 72 and 96 hours post-transfection. Virus production was measured by titering the virus-containing, cell-free supernatants on HeLa37 cells using single-hit infectivity assays described below.

### HIV single-hit infectivity assay

Short-term, single hit infectivity studies were performed as previously described [53]. HeLa37 cells were plated in a 48-well tray and triplicate wells were infected with a dual-tropic HIV-1_p256 _and serial dilutions of P-TEFb inhibitor for 40 hours. The cells were fixed with 75% acetone/25% H_2_O and immunostained for HIV-1 antigens using human anti-HIV serum (a gift from Dr. Jack Stapleton, Univ. of Iowa) and HRP-conjugated goat anti-human IgG followed by staining with 3-amino-9-ethylcarbazole (AEC). The HIV-1 antigen-positive cells were counted. Experiments were repeated at least three times with each drug concentration in triplicate. Results are represented as the means and standard errors of the mean of the percent of control values (the number of HIV-1 positive cells in the presence of P-TEFb inhibitors/the number of HIV-1 positive cells in untreated wells).

### Primary cell isolation, maintenance and infection with HIV

Human monocyte derived macrophages (MDMs) and peripheral blood lymphocytes (PBLs) cells were isolated from 350 ml of peripheral blood from healthy, HIV negative donors. Peripheral blood mononuclear cells (PBMCs) were isolated as previously described [53]. Briefly, PBMCs were separated by centrifugation in lymphocyte separation medium (ICN Biomedicals, Solon, Ohio). The separated PBMCs were placed on gelatin and fibronectin-coated flasks in order to separate monocytes from mononuclear cells. Adherent monocytes were lifted with EDTA, washed and plated at a density of 1 × 10^6 ^per well in 48-well trays for infectivity and cytotoxicity studies. Monocytes were differentiated for 5 days in DMEM with 10% FCS, 10% human serum and 1% penicillin/streptomycin in order to generate monocyte-derived macrophages prior to HIV infections and drug treatment. PBLs were treated with 5 μg/ml of phytohaemagglutinin (PHA) for 72 hours prior to HIV infection and drug treatment. PHA-treated PBLs were plated at a density of 1 × 10^6 ^per well in 48-well trays and maintained in RPMI 1640 with 10% FCS, 1% Penicillin/Streptomycin and 10 units/ml of recombinant IL-2. Viral infection was performed in MDMs and PBLs by adding 10,000 RT units of HIV-1_p256 _stock per 1 × 10^6 ^cells. During long term studies in primary cells, supernatants were collected at 4, 8, 12 and 16 days post-infection, frozen at -80°C until analyzed and media was refreshed. Inhibition of HIV replication by flavopiridol in PBLs was determined in 3 independent donors and each flavopirdol concentration was tested in triplicate. Inhibition of HIV replication by flavopiridol in MDMs was determined by pooling data from 3 independent donors. A minimum of 3 data points for each flavopiridol concentration was taken into account when generating the IC_50 _curve for MDMs.

### Cell viability assays

The impact of the P-TEFb inhibitors on cell viability was measured by ATPlite (Perkin Elmer). These cytotoxicity studies were performed as recommended by manufacturer utilizing a substrate solution that emits light in a manner proportional to the ATP present in each sample. Cells were plated in a 48-well format. Cells were treated with serial dilutions of the P-TEFb inhibitors and maintained for the indicated period of time. Mammalian cell lysis buffer was added to lyse the cells, followed by addition of the substrate solution. The amount of light produced in each well was measured in a TopCountR Microplate Scintillation and Luminescence Counter (Packard Instruments). Cytotoxicity experiments in HeLa37 cells were repeated at least three times with triplicates of each drug concentration. The results are represented as the means and standard errors of the mean of the percent of control values (the ATPLite values in the presence of P-TEFb inhibitors/the ATPLite values of untreated wells). Cytotoxicity studies in PBLs were performed in three independent donors and each flavopiridol concentration was tested in triplicate. The LD_50 _of flavopiridol in MDMs was determined by pooling data from 2 independent donors.

### P-TEFb kinase assays

Kinase reactions were carried out with recombinant, purified P-TEFb (Cdk9/cyclin T1) [6] and either DSIF subunit Spt5 or Pol II CTD as the substrate as previously described [55]. Kinase reactions contained 34 mM KCl, 20 mM HEPES pH 7.6, 7 mM MgCl_2_, 15 μM ATP, 1.3 μCi of [γ-^32^P]-ATP (Amersham) and 1 μg BSA. The reactions were incubated for 20 minutes at 30°C and stopped by addition of SDS-PAGE loading buffer. Reactions were resolved on a 7.5% SDS-PAGE gel. The dried gel was subjected to autoradiography. Quantitation was performed using an InstantImager (Packard) and data was normalized to the DMSO control. The data was fitted to a dose-response curve using TableCurve (Jandel Scientific) in order to determine the IC_50_.

### Glycerol gradient fractionation of cell lysates

HeLa cells were grown in 100 ml of DMEM with 10% FCS to a density of 4 × 10^5 ^cells/ml in spinner flasks. The cells were treated for 1 hour with no P-TEFb inhibitor or serial dilutions of DRB ranging from 0.1 to 10 μM. Cell lysates were prepared in Buffer A (10 mM KCl, 10 mM MgCl_2_, 10 mM HEPES, 1 mM EDTA, 1 mM DTT, 0.1% PMSF and EDTA-free complete protease inhibitor cocktail (Roche)) containing 150 mM NaCl and 0.5% NP-40. The lysates were clarified by centrifugation at 20,000 *g *for 10 minutes at 4°C. The supernatant was layered on top of a 5–45% glycerol gradient containing 150 mM NaCl. Gradients were spun at 190,000 *g *for 16 hours using a SW-55Ti rotor. The fractions were analyzed for the presence of P-TEFb complexes by immunoblotting with anti-cyclin T1 and anti-Cdk9 antibodies (Santa Cruz). Following incubation with the appropriate HRP-conjugated secondary antibodies, the blots were developed using SuperSignal DuraWest (Pierce). The western blots were imaged using a cooled CCD camera (UVP) and the amount of P-TEFb in the large and free form was quantitated using LabWorks 4.0 software.

### Separation of large and free forms of P-TEFb by differential salt extraction

HeLa37 and Jurkat cells were treated with serial dilutions of DRB, flavopiridol or seliciclib concentrations for 1 hour. The cytosolic extracts were prepared by resuspending the cells in 80 μl of Buffer A (10 mM KCl, 10 mM MgCl_2_, 10 mM HEPES, 1 mM EDTA, 1 mM DTT, 0.1% PMSF and EDTA-free complete protease inhibitor cocktail (Roche)) with 0.5% NP-40 for 10 minutes on ice. The nuclei were spun down at 5,000 *g *for 5 minutes and the supernatant was saved as the cytosolic extract (CE). The nuclei were washed once with 200 μl of Buffer A with 0.5% NP-40 and re-pelleted. The nuclei were resuspended in 80 μl of Buffer B (450 mM NaCl, 1.5 mM MgCl_2_, 20 mM HEPES, 0.5 mM EDTA, 1 mM DTT, 0.1% PMSF and EDTA-free complete protease inhibitor cocktail (Roche)) and incubated on ice for 10 minutes. The lysates were clarified by centrifugation at 20,000 *g *for 10 minutes. The supernatant was saved as the nuclear extract (NE). Western blotting was performed with one fifth of the samples and the fraction of Cdk9 and cyclin T1 in the cytosolic and nuclear extracts was determined by imaging the chemiluminescent signal using a cooled CCD camera (UVP). The signal was quantitated using LabWorks 4.0 software and the data fit to a logistic dose response curve using TableCurve (Jandel Scientific) to determine the IC_50 _for loss of the large, low salt extractable form of P-TEFb.

### Reverse transcriptase assays

Reverse transcriptase (RT) assays were performed on supernatants from HIV-1_p256 _infected cells as previously described [53]. Briefly, cell-free supernatant from infected cells were added to a mix containing 50 mM Tris (pH 7.8), 75 mM KCl, 2 mM DTT, 5 mM MgCl_2_, 0.05% NP-40, 5 μg poly(A), 4 μg poly(d) (T12-18) and 10 μCi/ml ^32^P-TTP. The mixture was incubated at 37°C for 3.5 hours and then blotted onto DE81 paper. The DE81 paper was washed 4 times with 3× SSPE and the amount of radioactivity that was incorporated into negative strand DNA was quantified with an InstantImager (Packard Instruments).

### Statistical analysis

All HIV infectivity and cytotoxicity data are represented as the percent of control values to allow comparisons of separate experiments. The mean of the values obtained in the infectivity studies were determined by averaging the individual experimental data points for each drug concentration. Error bars on graphs represent the calculated standard error for each drug dilution. Determination of IC_50 _and LD_50 _values was performed using TableCurve (Jandel Scientific).

## Abbreviations used

HIV-1, human immunodeficiency virus-1; P-TEFb, positive transcription elongation factor b; Cdk9, cyclin dependent kinase 9; CTD, carboxyl terminal domain; DSIF, DRB-sensitive inhibitory factor; HEXIM, hexamethylene bisacetamide-induced protein; Pol II, polymerase II; LTR, long terminal repeat; LD_50_, lethal dose_50_; IC_50_, inhibitory dose_50_; DRB, 5,6-dichloro-1-beta-D-ribofuranosylbenzimidazole; PBLs, peripheral blood lymphocytes; MDMs, monocyte derived macrophages; CE, cytosolic extracts; NP, nuclear pellet; PHA, phytohemagglutinin; IL-2, interleukin-2; RT, reverse transcriptase; HAART, highly active anti-retroviral therapy.

## Competing interests

This study was partially supported by Cyclacel Pharmaceuticals, Inc.

## Authors' contributions

SB was responsible for execution of the HIV-1 infectivity studies and preparation of the first draft of the manuscript. SAB was responsible for the execution of P-TEFb fractionation studies. SAB and JPP were responsible for execution of the kinase assays. VTN and OB contributed to the development of the salt extractions procedures for separation of free and large P-TEFb. DHP and WM are responsible for the overall design of the study and data interpretation. All authors were involved in revising the draft manuscript and approval of the final manuscript.
